# Supplementary material for: Mendelian randomization integrating GWAS and eQTL data reveals genetic determinants of complex and clinical traits
Source: Nat Commun. 2019 Jul 24;10:3300. doi: 10.1038/s41467-019-10936-0 (PMC6656778; doi:10.1038/s41467-019-10936-0)
Supplement: Supplementary file 1 — Supplementary Information [file 41467_2019_10936_MOESM1_ESM.pdf]

Supplementary Information

Mendelian Randomization integrating GWAS and eQTL data reveals genetic determinants of complex and clinical traits

Porcu et al.

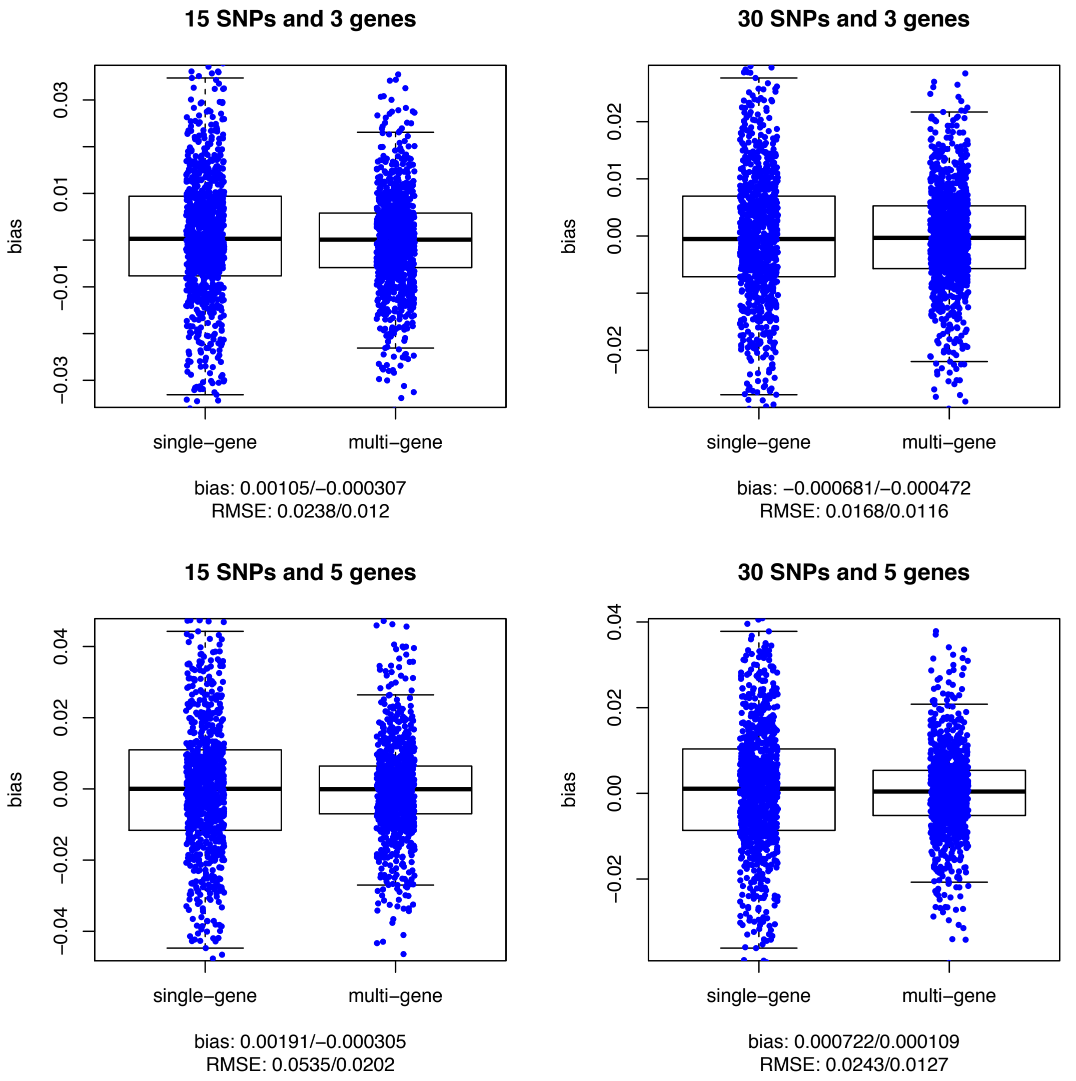

Supplementary Figure 1. Simulation analyses results. We simulated four regions containing 15 and 30 SNPs and 3 and 5 genes assuming a degree of pleiotropy from a Poisson distribution (λ=0.2, i.e. λ is the mean of the number of genes affected by the SNP) and for each scenario we calculated the bias (i.e.: the difference between the observed and the true value) and the Root Mean Squared Error (RMSE) for the single- and multi-gene MR approach.

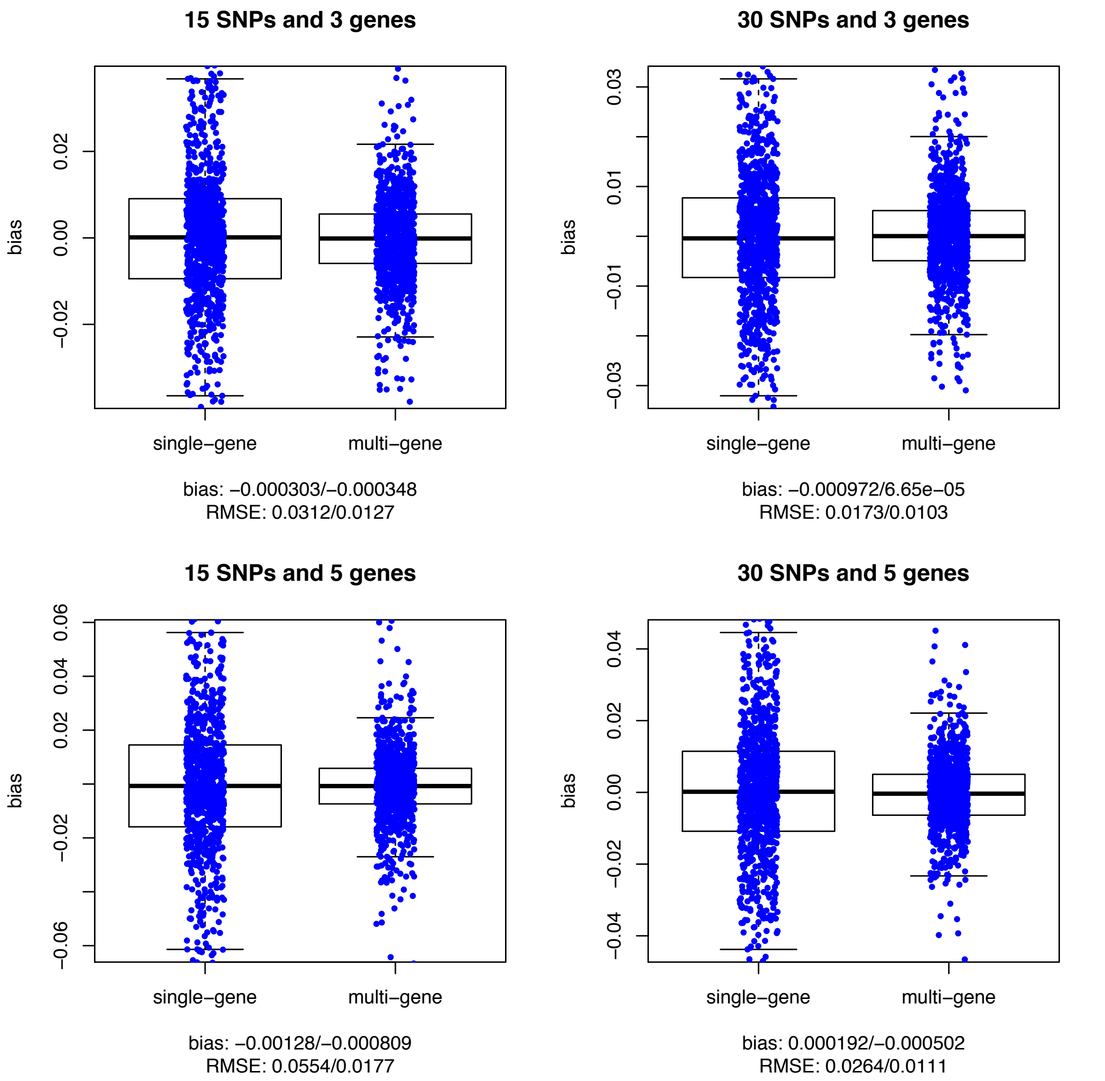

Supplementary Figure 2. Simulation analyses results. We simulated four regions containing 15 and 30 SNPs and 3 and 5 genes assuming a degree of pleiotropy from a Poisson distribution (λ=0.4, i.e. λ is the mean of the number of genes affected by the SNP) and for each scenario we calculated the bias (i.e.: the difference between the observed and the true value) and the Root Mean Squared Error (RMSE) for the single- and multi-gene MR approach.

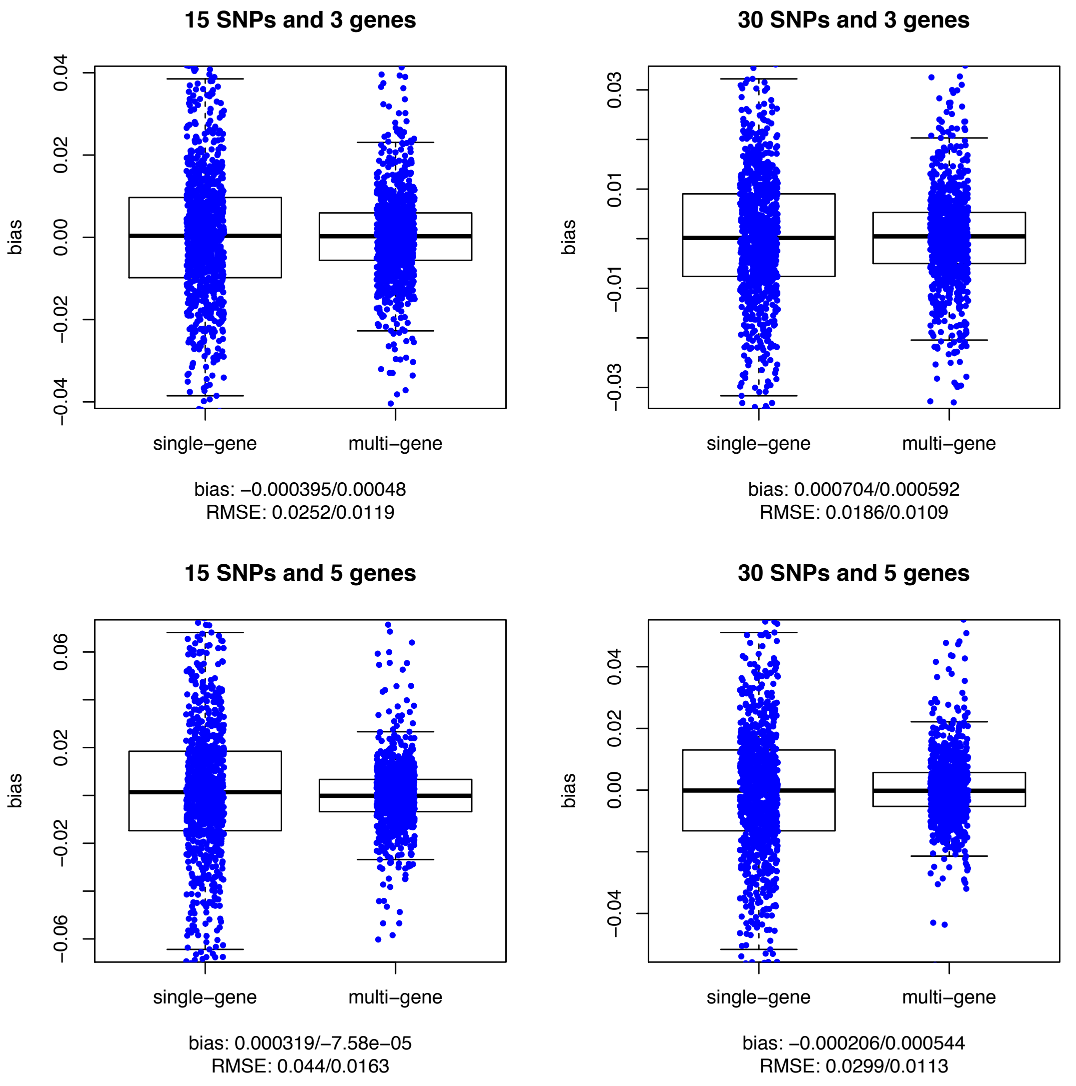

Supplementary Figure 3. Simulation analyses results. We simulated four regions containing 15 and 30 SNPs and 3 and 5 genes assuming a degree of pleiotropy from a Poisson distribution (λ=0.6, i.e. λ is the mean of the number of genes affected by the SNP) and for each scenario we calculated the bias (i.e.: the difference between the observed and the true value) and the Root Mean Squared Error (RMSE) for the single- and multi-gene MR approach.

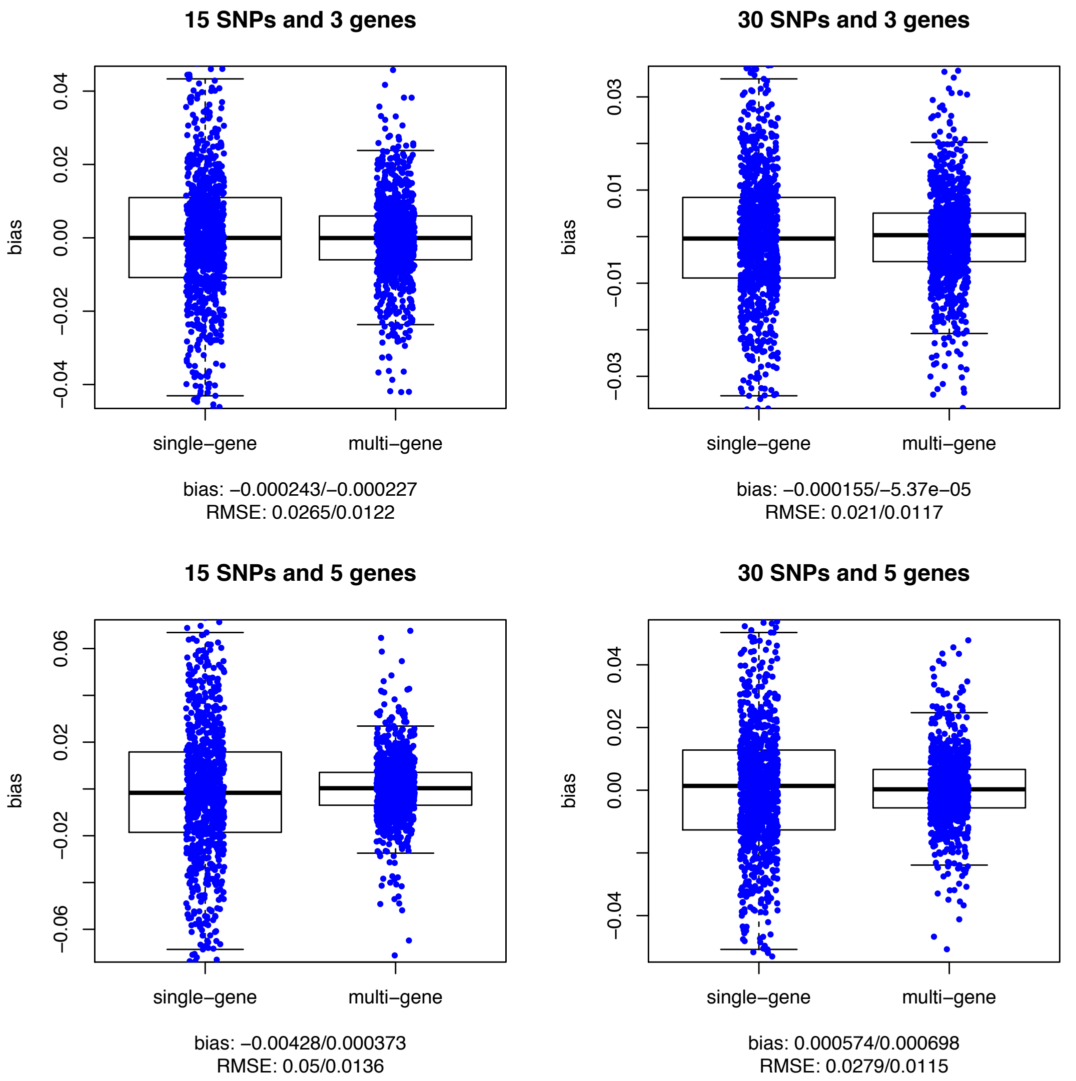

Supplementary Figure 4. Simulation analyses results. We simulated four regions containing 15 and 30 SNPs and 3 and 5 genes assuming a degree of pleiotropy from a Poisson distribution (λ=0.8, i.e. λ is the mean of the number of genes affected by the SNP) and for each scenario we calculated the bias (i.e.: the difference between the observed and the true value) and the Root Mean Squared Error (RMSE) for the single- and multi-gene MR approach.

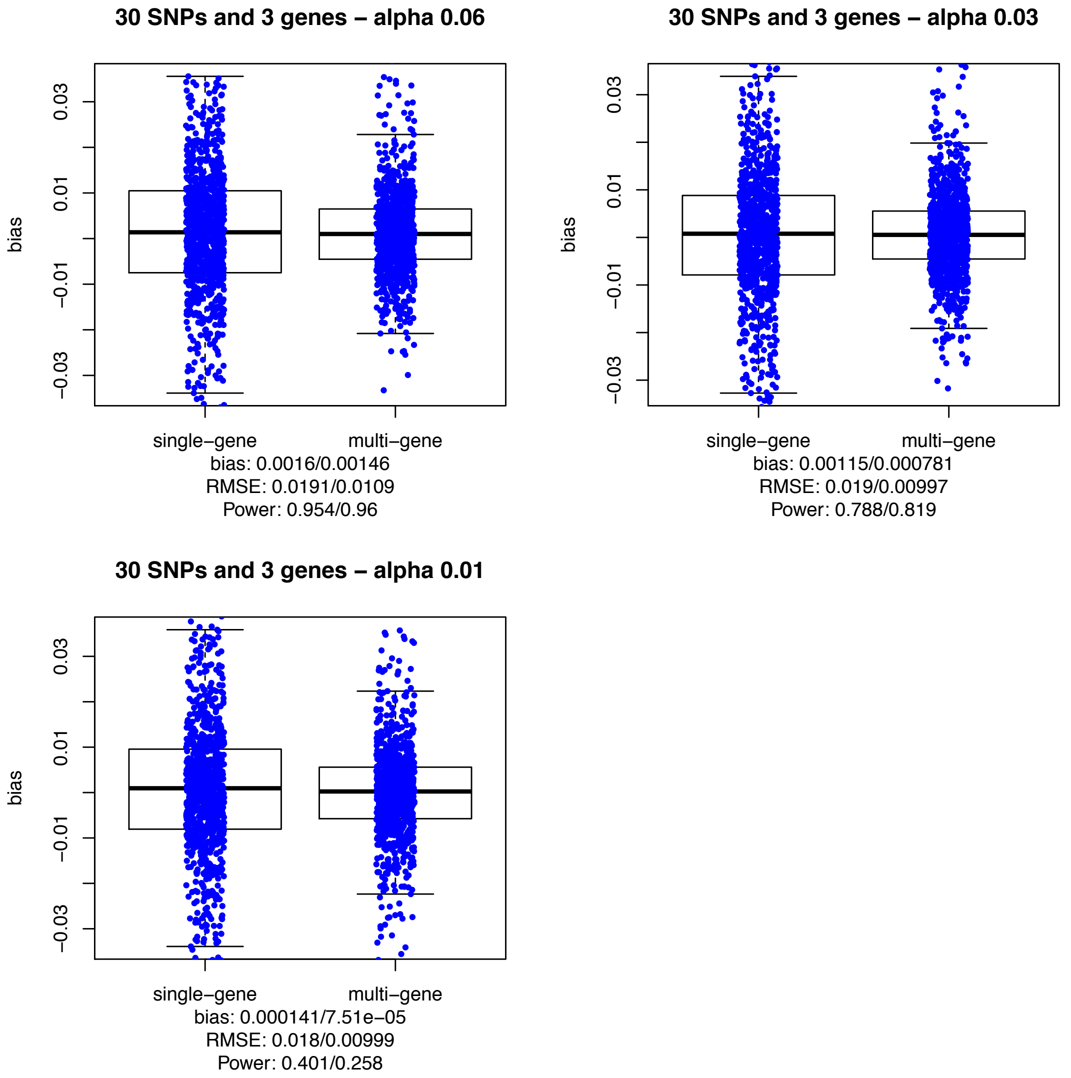

Supplementary Figure 5. Simulation analyses results. We simulated three different causal effects of the gene expression on the trait (α=0.06, 0.03 and 0.01) in a region containing 30 SNPs and 3 genes assuming a degree of pleiotropy from a Poisson distribution (λ=0.6, i.e. λ is the mean of the number of genes affected by the SNP). We calculated the power as the ratio of number of times we obtained $P_{\mathbf{MR}}$<0.05 and the number of simulations (1,000).

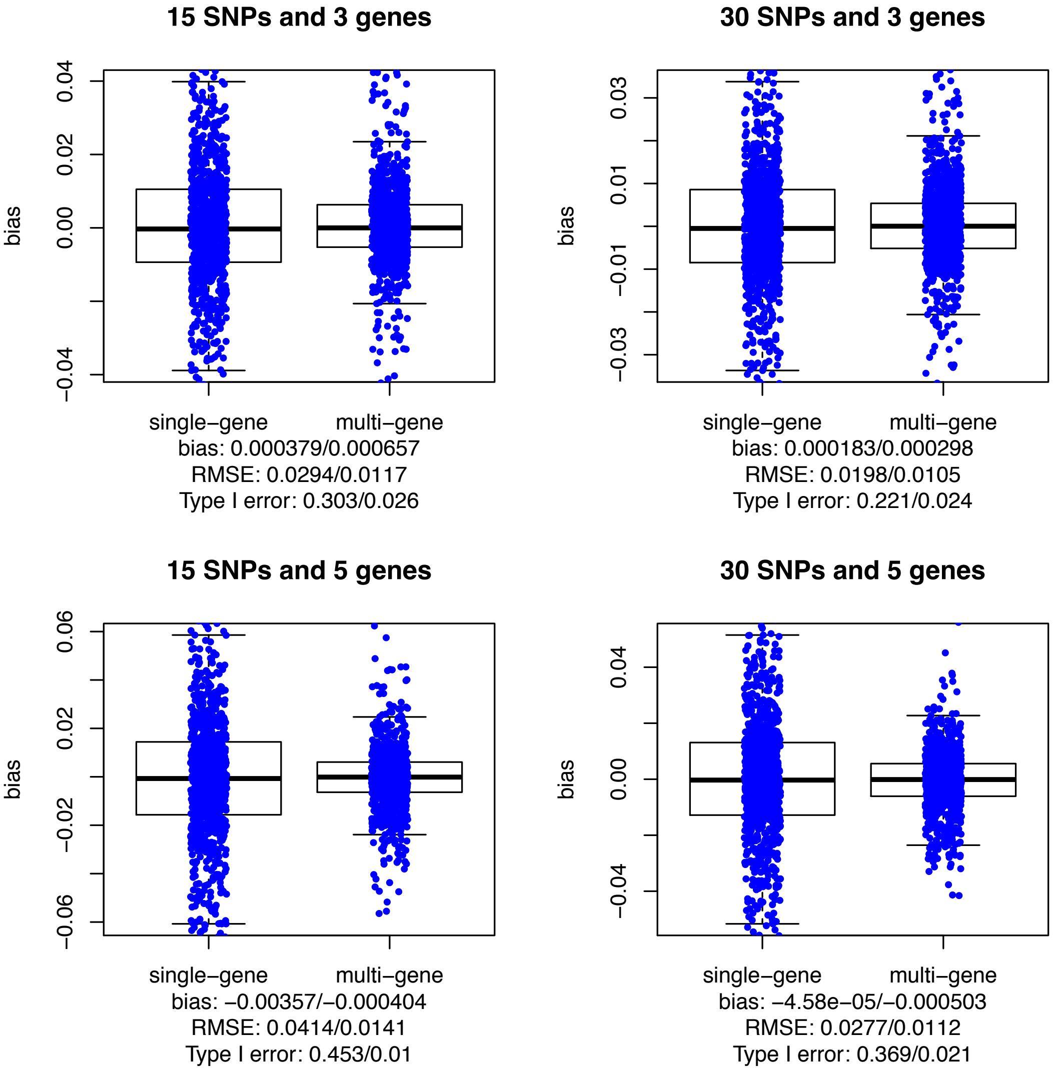

Supplementary Figure 6. Simulation analyses results. We assume a zero causal effect of the gene expression on the trait (α=0) in four regions containing 15 and 30 SNPs and 3 and 5 genes assuming a degree of pleiotropy from a Poisson distribution (λ=0.6, i.e. λ is the mean of the number of genes affected by the SNP). We calculated the type I error as the ratio of number of times we obtained $P_{\mathbf{MR}}$<0.05 and the number of simulations (1,000).

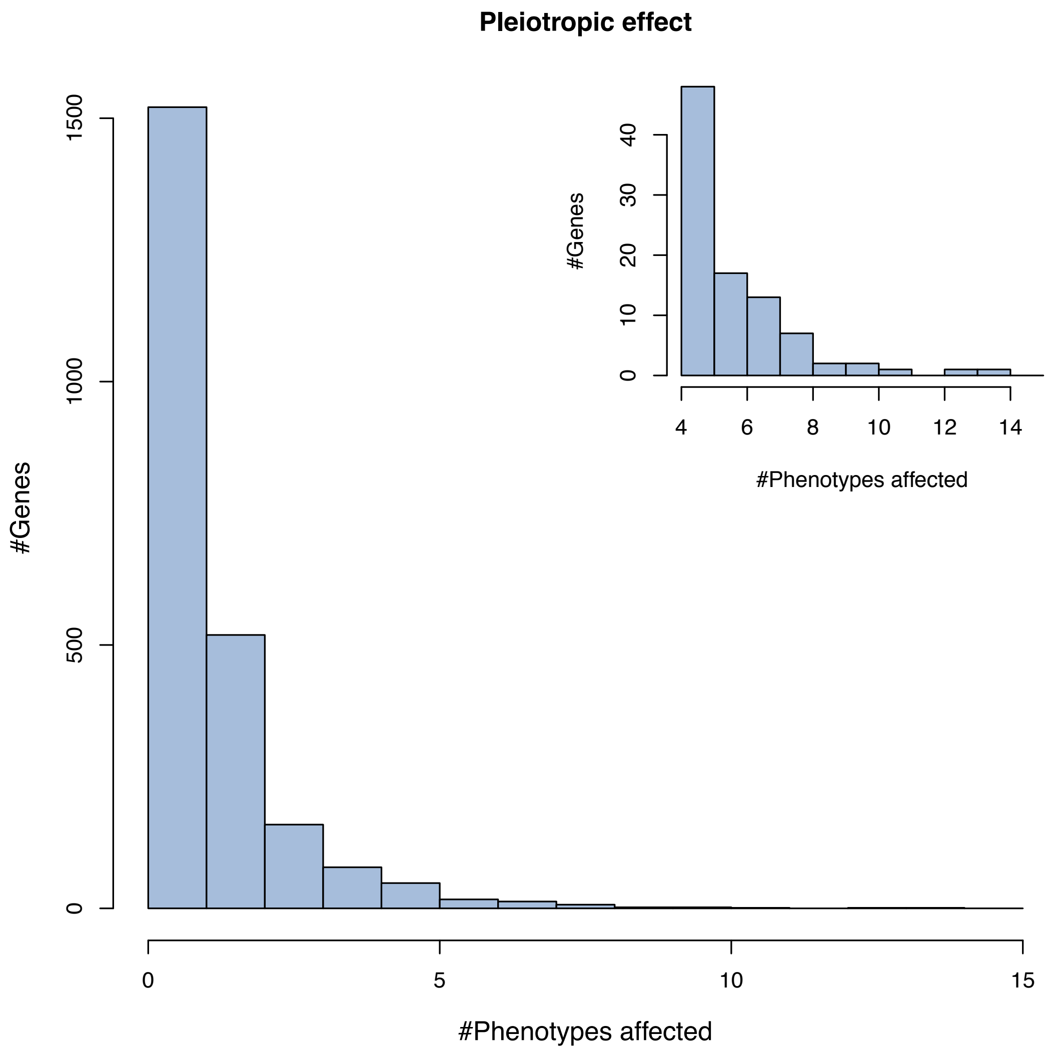

Supplementary Figure 7. Number of genes associated with multiple phenotypes.

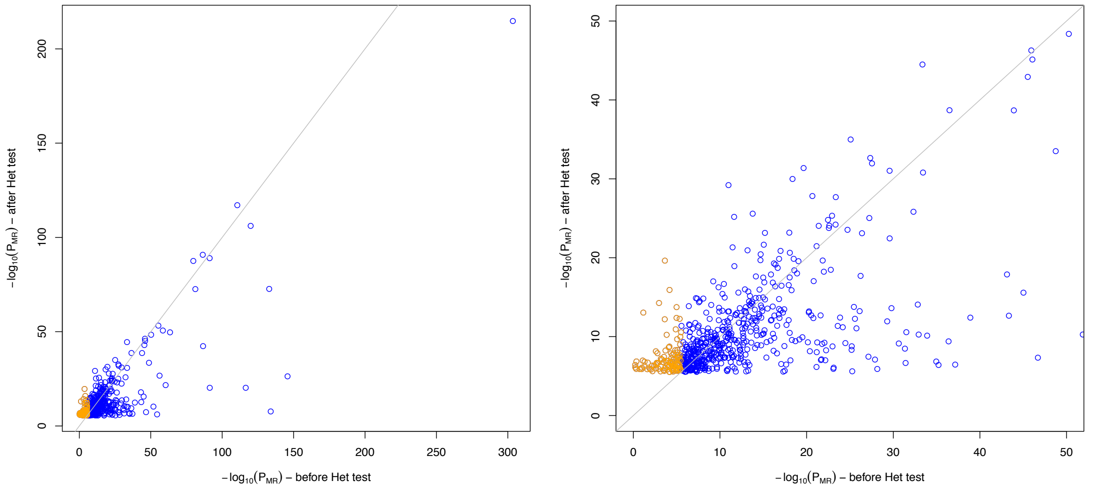

Supplementary Figure 8. P-values of genes showing heterogeneity. Shown are the results from multi-instrument and multi-exposure MR analysis after the outlier removal (y-axis) compared with those (x-axis) before the outlier removal. In orange are the genes that pass the P-value threshold 3×10^{-06} only after the outlier removal. The grey line represents the identity line. (Plot on the right is the zoom in of the plot on the left)

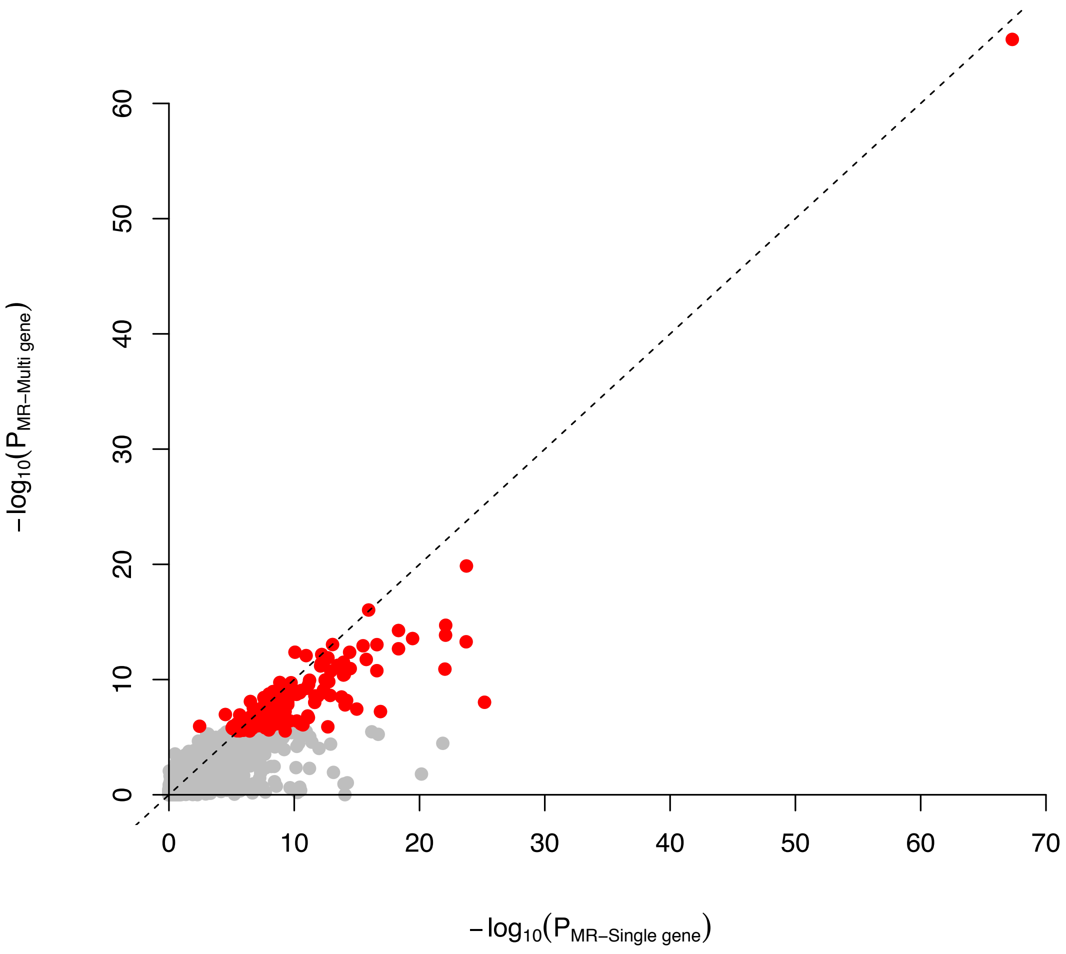

Supplementary Figure 9. Multi- vs Single-exposure MR. Shown are the results from multi-instrument and multi-exposure MR analysis (y-axis) compared with those from the multi-instruments and single-exposure MR analysis (x-axis) on menarche. In grey are the genes that do not pass the P-value threshold $3x10^{-06}$ in the multi-instrument and multi-exposure MR analysis. The dotted line represents the identity line.

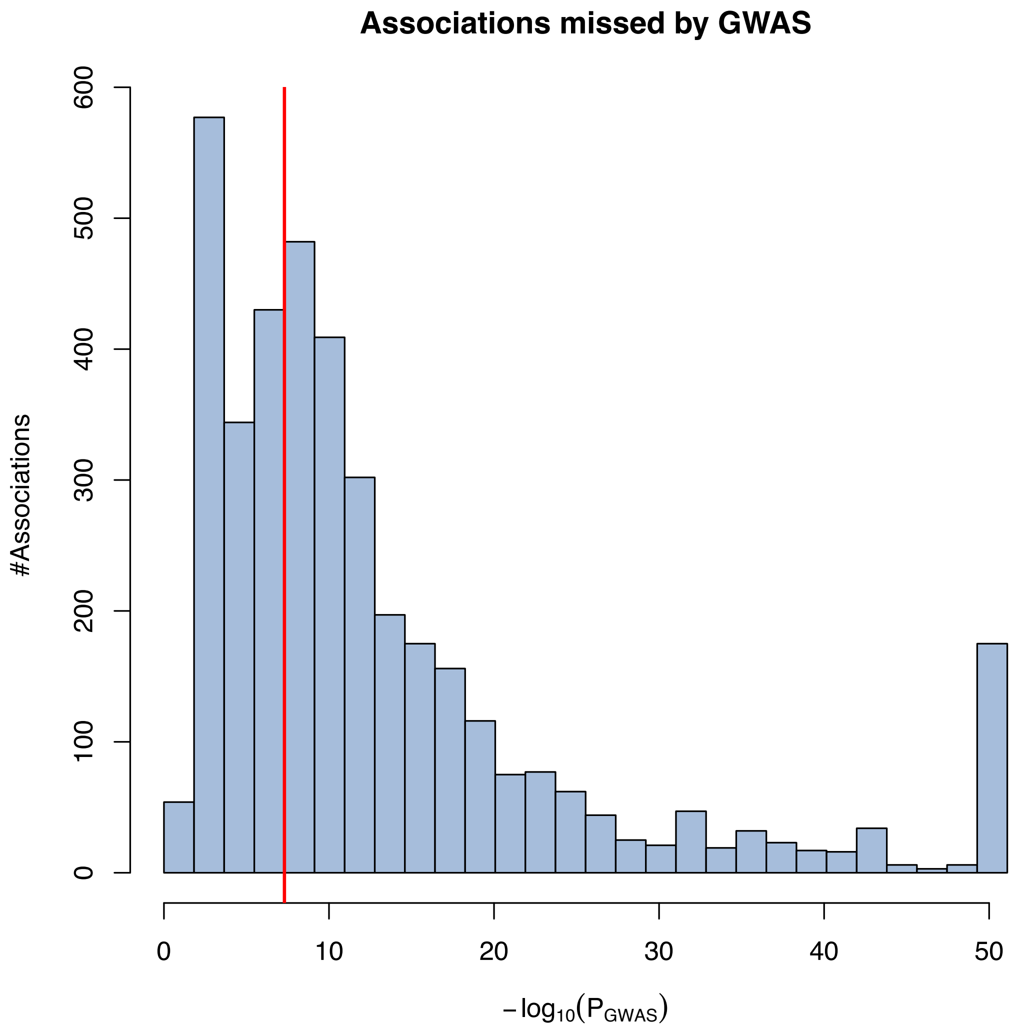

Supplementary Figure 10. Distribution of topSNP association P-values at reported putative causal genes. For each gene, the best SNP pvalue at +/-500kb is reported. The red line represents genome-wide significance.

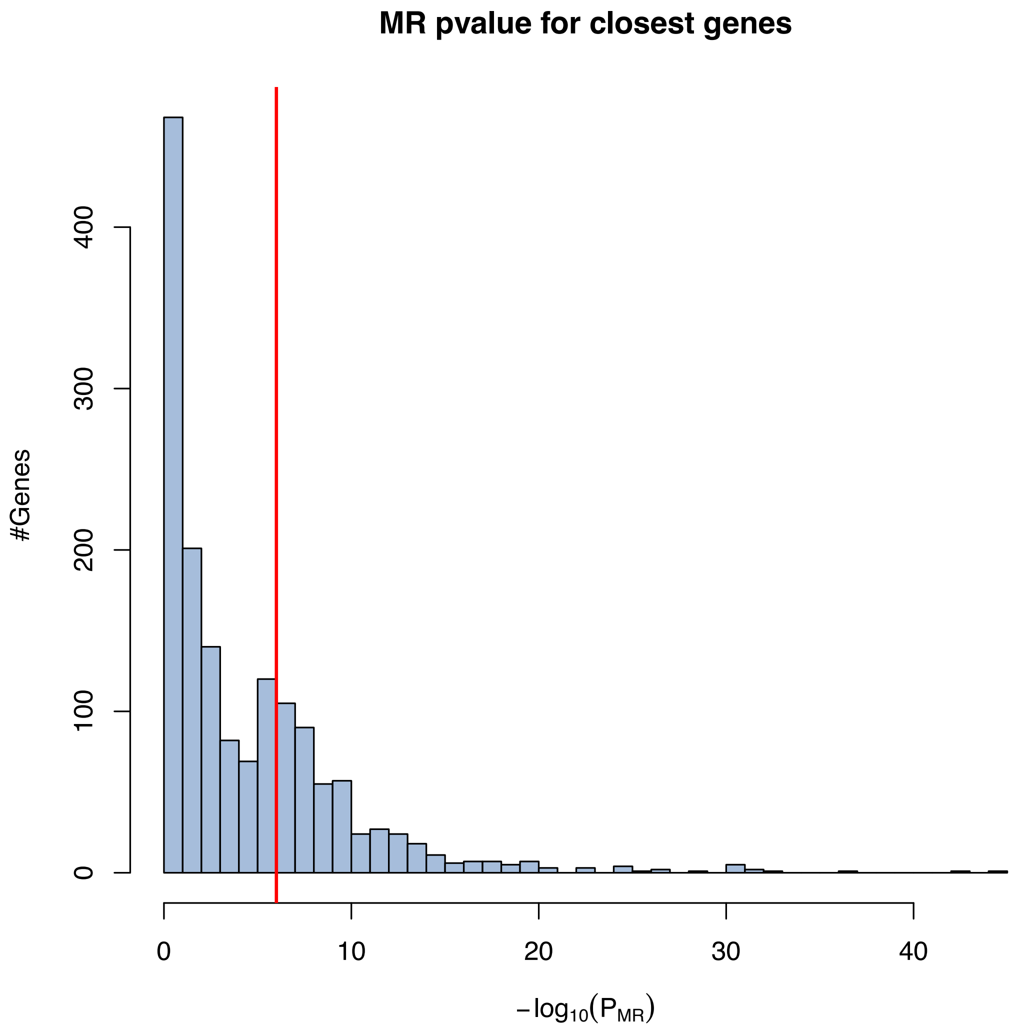

Supplementary Figure 11. Distribution of the MR-pvalues for the nearest genes. For the 1,125 MR significant regions harbouring at least one genome-wide significant SNP, we plotted the MR-pvalue of the gene closest to the top SNP in the region. The red line represents MR significance threshold.

A

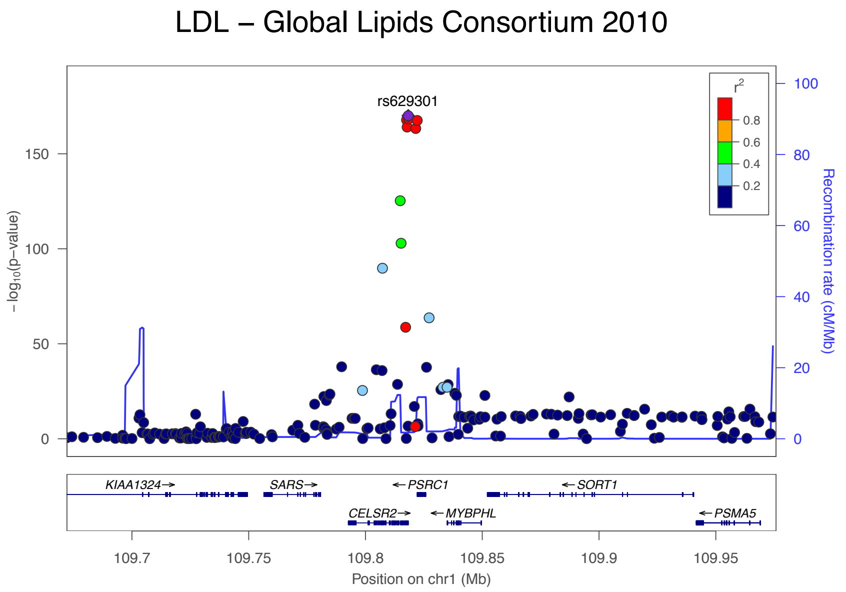

B

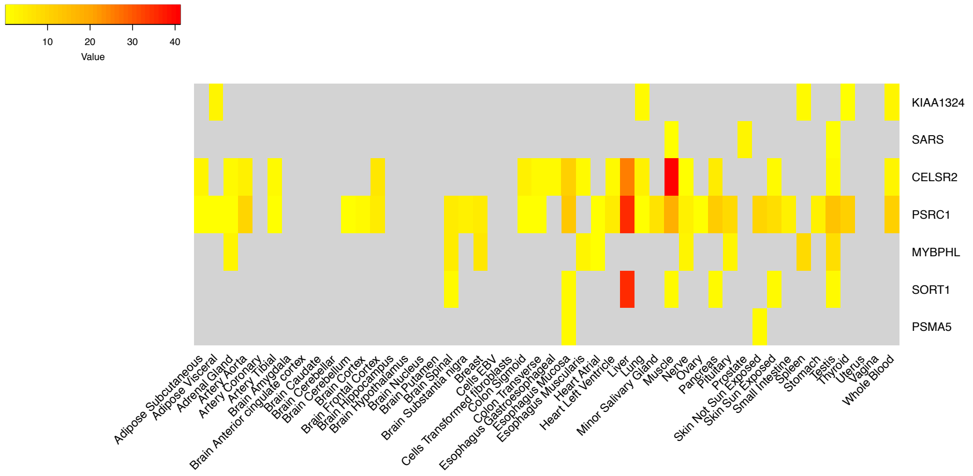

Supplementary Figure 12. Tissue-specific effects of *SORT1* on LDL. **a.** Regional association plot showing genome-wide significant locus for LDL at *SORT1* gene region. Representation of the single-SNP association strength (y axis shows the –log10 p-value) versus the genomic positions (on hg19/GRCh37 genomic build; x axis) around the most significant SNP, which is indicated with a purple dot. Other SNPs in the region are color coded to reflect their LD with the top SNP, as in the left inset (taken from pairwise r2 values calculated on 1000 Genomes Project phase 3 haplotypes). Genes and the position of exons, as well as the direction of transcription, are noted in lower boxes. This plot was drawn using the standalone version of the LocusZoom package (Pruim et al., 2010). **b.** Tissue-specific causal effects. Rows list genes and columns list tissues. Darker points correspond to higher association. The genes highlighted in grey were not tested.

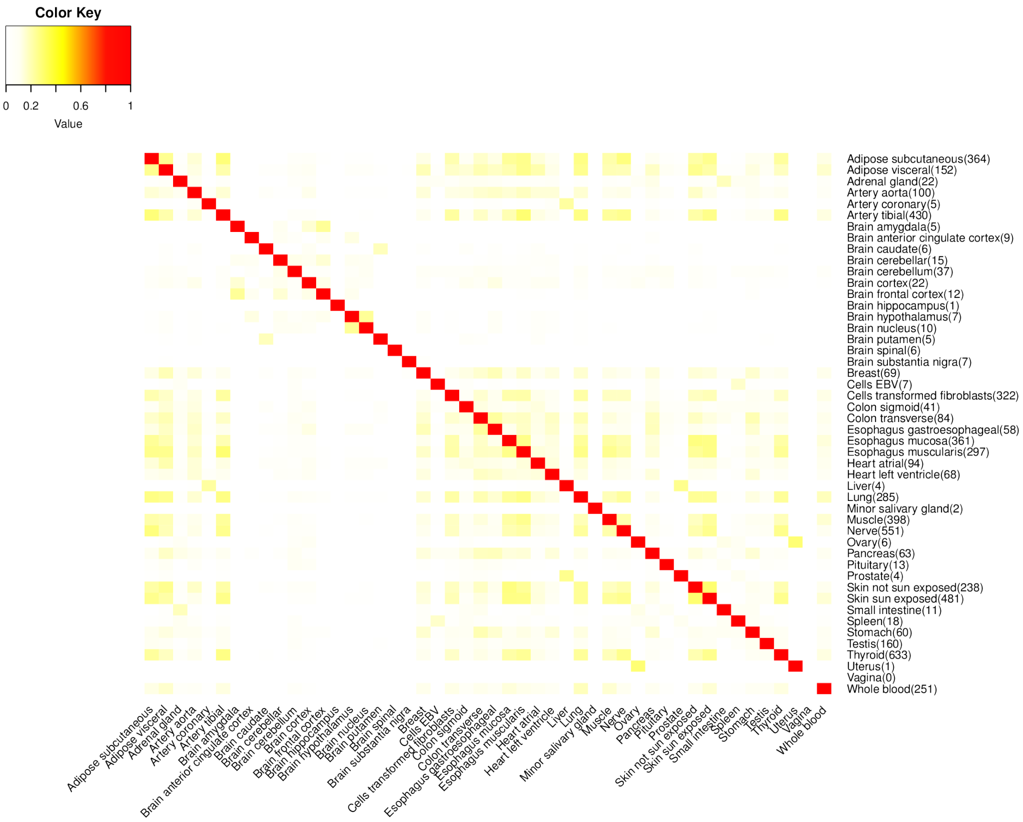

Supplementary Figure 13. Estimates of eGenes sharing among the 48 GTEx tissues. The heatmap represents the portion of eGenes, which have at least 4 instrumental variables, shared among the tissues for Crohn's disease. Each cell shows the portion of shared eGenes calculated using the Jaccard index.

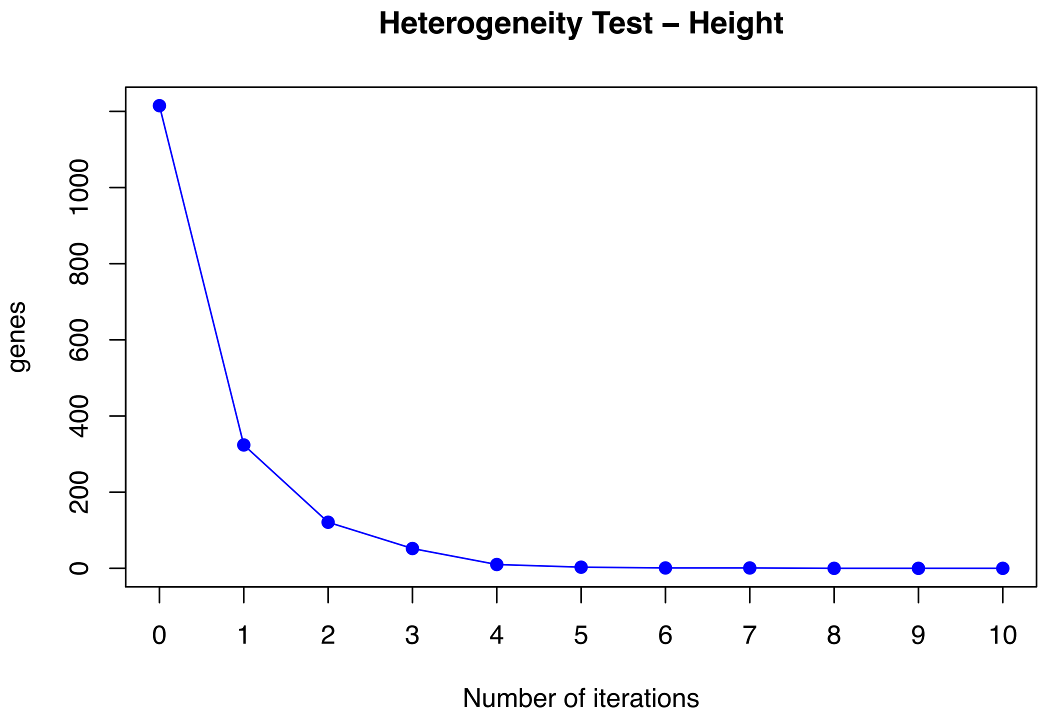

Supplementary Figure 14. The number of genes with significant P-value in heterogeneity test. The number of genes with $P_{het}<10^{-4}$ for height. At each iteration, for the genes showing significant pleiotropy, we remove the most pleiotropic SNP and then repeat the test. We observed that after 3 iterations we corrected more than 90% of the genes and after 5 we reach a plateau.

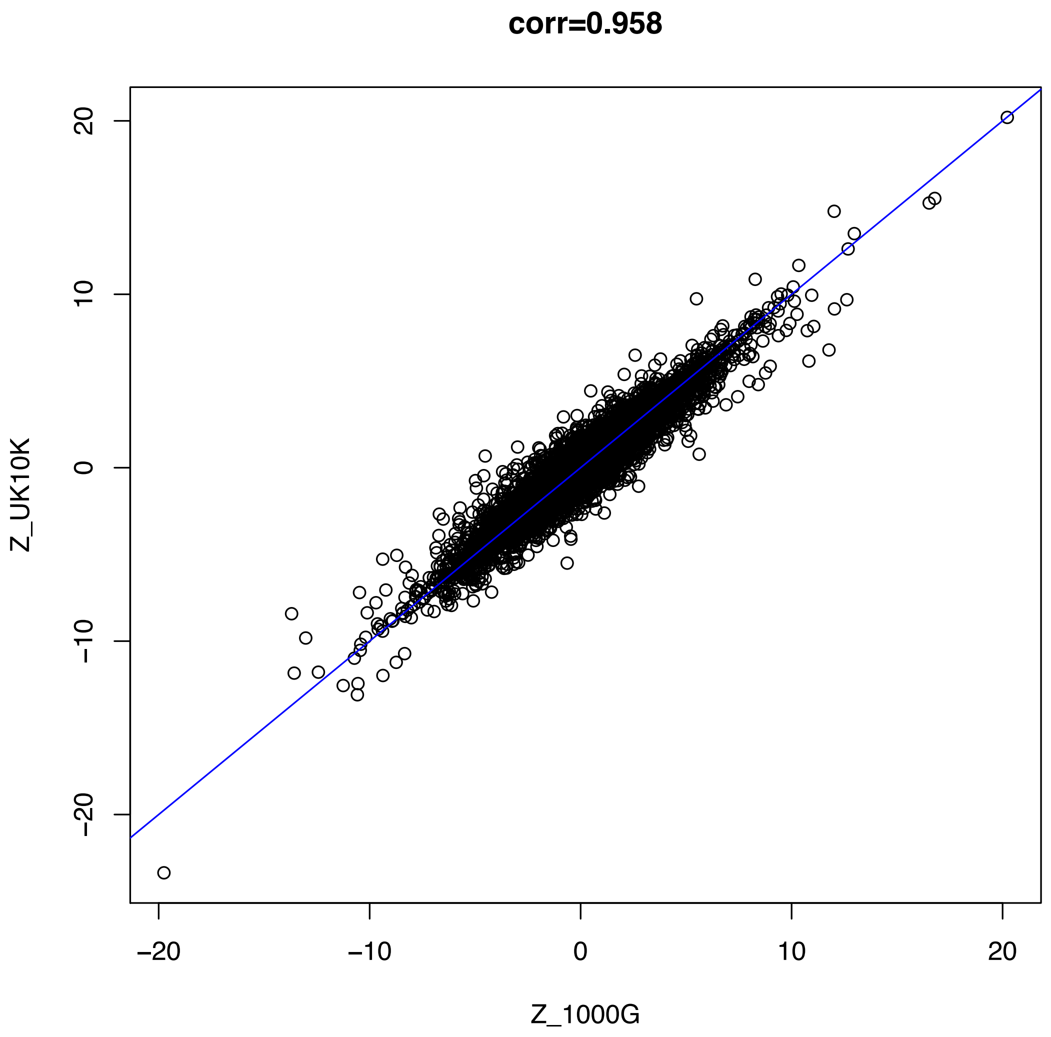

Supplementary Figure 15. UK10K vs 1000g-EUR reference panel. Shown are the results (Z-scores) from multi-instrument and multi-exposure MR analysis applied using UK10K reference panel (y-axis) compared with those from the same approach but using 1000G-EUR on height. The blue line represents the identity line.

Supplementary Table 1. Phenotypes analyzed in this study

TRAIT	Abbreviation	N (case/control)	Significant genes	missed by GWAS	%missed by GWAS	N tastable gene	Ref
Alzheimer's Disease	AD	25,580 / 48,466	17	10	58.8	14,772	Lambert et al (2013) [1]
Amyotrophic Lateral Sclerosis	ALS	12,577 / 23,475	8	7	87.5	14,849	van Rheenen et al (2016) [2]
Basophils	BASO	173,480	61	16	26.2	15,086	Astle et al (2016) [3]
Body Mass Index	BMI	339,224	31	6	19.4	13,864	Locke et al (2015) [4]
Corononary Artery Disease	CAD	60,801 / 123,504	27	19	70.4	15,041	Nikpay et al (2015) [5]
Crohn's Disease	CD	5,956 / 14,927	61	16	26.2	15,147	Liu et al (2015) [6]
Childhood Body Mass Index	cBMI	35,668	24	19	79.2	13,859	Felix et al (2015) [7]
Depressive syntoms	DEPRESSION	161,460	1	1	100.0	14,686	Okbay et al (2016) [8]
Diastolic blood pressure	DP	69,899	13	7	53.9	13,854	International Consortium for Blood Pressure Genome-Wide Association Studies (2011) [9]
Educational attainment	EDU	111,483	76	37	48.7	15,129	Okbay et al (2016) [10]
Eosinophils	EO	173,480	175	38	21.7	15,085	Astle et al (2016) [3]
Fasting Glucose	FG	96,496	5	1	20.0	13,973	Manning et al (2012) [11]
Fasting Insulin	FI	96,496	5	1	20.0	13,984	Manning et al (2012) [11]
High-Density Lipoprotein	HDL	99,900	204	162	79.4	13,889	Teslovich et al (2010) [12]
Height	HEIGHT	253,288	374	43	11.5	13,849	Wood et al (2014) [13]
Hemoglobin	HGB	173,480	108	12	11.1	15,086	Astle et al (2016) [3]
Heart Rate	HR	181,171	11	6	54.5	13,836	den Hoed et al (2014) [14]
Inflammatory Bowel Disease	IBD	12,882 / 21770	69	20	29.0	15,154	Liu et al (2015) [6]
Insomnia	INSOMNIA	113,006	3	3	100.0	15,075	Hammerschlag et al (2017) [15]
Intelligence	IQ	78,308	17	9	52.9	15,076	Sniekers et al (2017) [16]
Low-Density Lipoprotein	LDL	95,454	177	150	84.7	13,889	Teslovich et al (2010) [12]
Lymphocytes	LYMPH	173,480	179	35	19.6	15,086	Astle et al (2016) [3]
Mean Corpuscolar Volume	MCV	173,480	214	20	9.3	15,086	Astle et al (2016) [3]
Mean arterial blood pressure	MABP	29,182	9	5	55.6	13,830	International Consortium for Blood Pressure Genome-Wide Association Studies (2011) [9]
Menarche	MENARCHE	329,345	155	37	23.9	15,029	Day et al (2017) [17]
Menopause	MENOPAUSE	69,360	53	16	30.2	13,748	Day et al (2015) [18]

Monocytes	MONO	173,480	185	30	16.2	15,086	Astle et al (2016) [3]
Mean Platelet Volume	MPV	173,480	194	36	18.6	15,086	Astle et al (2016) [3]
Neuroticism	NEUROTICISM	170,911	39	13	33.3	14,685	Okbay et al (2016) [8]
Neutrophils	NEUT	173,480	175	34	19.4	15,086	Astle et al (2016) [3]
Platelets	PLT	173,480	209	30	14.4	15,085	Astle et al (2016) [3]
Pulse blood pressure	PBP	74,079	8	8	100.0	13,856	International Consortium for Blood Pressure Genome-Wide Association Studies (2011) [9]
Rheumatoid Arthritis	RA	29,880 / 73,758	41	18	43.9	14,508	Okada et al (2014) [19]
Red Blood Cells	RBC	173,480	180	28	15.6	15,086	Astle et al (2016) [3]
Reticulocytes	RET	173,480	156	26	16.7	15,086	Astle et al (2016) [3]
Schizophrenia	SCZ	36,989 / 113,075	122	64	52.5	15,037	Schizophrenia Working Group of the Psychiatric Genomics Consortium et al (2014) [20]
Subjective Well Being	SWB	298,420	5	1	20.0	13,568	Okbay et al (2016) [8]
Systolic blood pressure	SBP	69,909	14	11	78.6	13,853	International Consortium for Blood Pressure Genome-Wide Association Studies (2011) [9]
Type 2 Diabetes	T2D	26,676 / 132,532	44	35	79.6	15,071	Scott et al (2017) [21]
Total Cholesterol	TC	100,184	207	169	81.6	13,887	Teslovich et al (2010) [12]
Triglycerides	TG	96,598	178	154	88.8	13,887	Teslovich et al (2010) [12]
Ulcerative Colitis	UC	6,968 / 20,464	35	15	42.9	15,148	Liu et al (2015) [6]
Waist Hip Ratio (BMI-adjusted)	WHRadjBMI	224,459	44	27	61.4	13,855	Shungin et al (2015) [22]

Supplementary Table 2. Sample size of GTEx tissues

Tissue	Number of RNASeq and Genotyped samples
Adipose – Subcutaneous	385
Adipose - Visceral (Omentum)	313
Adrenal Gland	175
Artery – Aorta	267
Artery – Coronary	152
Artery – Tibial	388
Brain – Amygdala	88
Brain - Anterior cingulate cortex (BA24)	109
Brain - Caudate (basal ganglia)	144
Brain - Cerebellar Hemisphere	125
Brain – Cerebellum	154
Brain – Cortex	136
Brain - Frontal Cortex (BA9)	118
Brain – Hippocampus	111
Brain – Hypothalamus	108
Brain - Nucleus accumbens (basal ganglia)	130
Brain - Putamen (basal ganglia)	111
Brain - Spinal cord (cervical c-1)	83
Brain - Substantia nigra	80
Breast - Mammary Tissue	251
Cells - EBV-transformed lymphocytes	117
Cells - Transformed fibroblasts	300
Colon – Sigmoid	203
Colon – Transverse	246
Esophagus - Gastroesophageal Junction	213
Esophagus – Mucosa	358
Esophagus – Muscularis	335
Heart - Atrial Appendage	264
Heart - Left Ventricle	272
Liver	153
Lung	383
Minor Salivary Gland	85
Muscle – Skeletal	491
Nerve – Tibial	361
Ovary	122
Pancreas	220
Pituitary	157
Prostate	132
Skin - Not Sun Exposed (Suprapubic)	335

Skin - Sun Exposed (Lower leg)	414
Small Intestine - Terminal Ileum	122
Spleen	146
Stomach	237
Testis	225
Thyroid	399
Uterus	101
Vagina	106
Whole Blood	369

References

1. Lambert, J.C., et al., *Meta-analysis of 74,046 individuals identifies 11 new susceptibility loci for Alzheimer's disease.* Nat Genet, 2013. **45**(12): p. 1452-8.
2. van Rheenen, W., et al., *Genome-wide association analyses identify new risk variants and the genetic architecture of amyotrophic lateral sclerosis.* Nat Genet, 2016. **48**(9): p. 1043-8.
3. Astle, W.J., et al., *The Allelic Landscape of Human Blood Cell Trait Variation and Links to Common Complex Disease.* Cell, 2016. **167**(5): p. 1415-1429 e19.
4. Locke, A.E., et al., *Genetic studies of body mass index yield new insights for obesity biology.* Nature, 2015. **518**(7538): p. 197-206.
5. Nikpay, M., et al., *A comprehensive 1,000 Genomes-based genome-wide association meta-analysis of coronary artery disease.* Nat Genet, 2015. **47**(10): p. 1121-1130.
6. Liu, J.Z., et al., *Association analyses identify 38 susceptibility loci for inflammatory bowel disease and highlight shared genetic risk across populations.* Nat Genet, 2015. **47**(9): p. 979-986.
7. Felix, J.F., et al., *Genome-wide association analysis identifies three new susceptibility loci for childhood body mass index.* Hum Mol Genet, 2016. **25**(2): p. 389-403.
8. Okbay, A., et al., *Genetic variants associated with subjective well-being, depressive symptoms, and neuroticism identified through genome-wide analyses.* Nat Genet, 2016. **48**(6): p. 624-33.
9. International Consortium for Blood Pressure Genome-Wide Association, S., et al., *Genetic variants in novel pathways influence blood pressure and cardiovascular disease risk.* Nature, 2011. **478**(7367): p. 103-9.
10. Okbay, A., et al., *Genome-wide association study identifies 74 loci associated with educational attainment.* Nature, 2016. **533**(7604): p. 539-42.
11. Manning, A.K., et al., *A genome-wide approach accounting for body mass index identifies genetic variants influencing fasting glycemic traits and insulin resistance.* Nat Genet, 2012. **44**(6): p. 659-69.

12. Teslovich, T.M., et al., *Biological, clinical and population relevance of 95 loci for blood lipids.* Nature, 2010. **466**(7307): p. 707-13.
13. Wood, A.R., et al., *Defining the role of common variation in the genomic and biological architecture of adult human height.* Nat Genet, 2014. **46**(11): p. 1173-86.
14. den Hoed, M., et al., *Identification of heart rate-associated loci and their effects on cardiac conduction and rhythm disorders.* Nat Genet, 2013. **45**(6): p. 621-31.
15. Hammerschlag, A.R., et al., *Genome-wide association analysis of insomnia complaints identifies risk genes and genetic overlap with psychiatric and metabolic traits.* Nat Genet, 2017. **49**(11): p. 1584-1592.
16. Sniekers, S., et al., *Genome-wide association meta-analysis of 78,308 individuals identifies new loci and genes influencing human intelligence.* Nat Genet, 2017. **49**(7): p. 1107-1112.
17. Day, F.R., et al., *Genomic analyses identify hundreds of variants associated with age at menarche and support a role for puberty timing in cancer risk.* Nat Genet, 2017. **49**(6): p. 834-841.
18. Day, F.R., et al., *Large-scale genomic analyses link reproductive aging to hypothalamic signaling, breast cancer susceptibility and BRCA1-mediated DNA repair.* Nat Genet, 2015. **47**(11): p. 1294-1303.
19. Okada, Y., et al., *Genetics of rheumatoid arthritis contributes to biology and drug discovery.* Nature, 2014. **506**(7488): p. 376-81.
20. Schizophrenia Working Group of the Psychiatric Genomics, C., *Biological insights from 108 schizophrenia-associated genetic loci.* Nature, 2014. **511**(7510): p. 421-7.
21. Scott, R.A., et al., *An Expanded Genome-Wide Association Study of Type 2 Diabetes in Europeans.* Diabetes, 2017. **66**(11): p. 2888-2902.
22. Shungin, D., et al., *New genetic loci link adipose and insulin biology to body fat distribution.* Nature, 2015. **518**(7538): p. 187-196.
