# Supplementary material for: Mendelian randomization integrating GWAS and eQTL data reveals genetic determinants of complex and clinical traits
Source: Nat Commun. 2019 Jul 24;10:3300. doi: 10.1038/s41467-019-10936-0 (PMC6656778; doi:10.1038/s41467-019-10936-0)
Supplement: Supplementary file 8 — Description of Additional Supplementary Files [file 41467_2019_10936_MOESM8_ESM.docx]

**Title:** Supplementary Data 1.
**Description:** List of 3913 significant trait-gene associations

**Title:** Supplementary Data 2.
**Description:** List of significant results for Coronary Artery Diseases in 48 GTEx tissues

**Title:** Supplementary Data 3.
**Description:** List of significant results for Crohn's Disease in 48 GTEx tissues

**Title:** Supplementary Data 4.
**Description:** List of significant results for LDL in 48 GTEx tissues

**Title:** Supplementary Data 5.
**Description:** List of significant results for Type2 Diabetes in 48 GTEx tissues
